# Flow-Dependent Mass Transfer May Trigger Endothelial Signaling Cascades

**DOI:** 10.1371/journal.pone.0035260

**Published:** 2012-04-27

**Authors:** Prashanthi Vandrangi, Martha Sosa, John Y.-J. Shyy, Victor G. J. Rodgers

**Affiliations:** 1 Department of Bioengineering, University of California Riverside, Riverside, California, United States of America; 2 Division of Biomedical Sciences, University of California Riverside, Riverside, California, United States of America; Ohio State University, United States of America

## Abstract

It is well known that fluid mechanical forces directly impact endothelial signaling pathways. But while this general observation is clear, less apparent are the underlying mechanisms that initiate these critical signaling processes. This is because fluid mechanical forces can offer a direct mechanical input to possible mechanotransducers as well as alter critical mass transport characteristics (i.e., concentration gradients) of a host of chemical stimuli present in the blood stream. However, it has recently been accepted that mechanotransduction (direct mechanical force input), and not mass transfer, is the fundamental mechanism for many hemodynamic force-modulated endothelial signaling pathways and their downstream gene products. This conclusion has been largely based, indirectly, on accepted criteria that correlate signaling behavior and shear rate and shear stress, relative to changes in viscosity. However, in this work, we investigate the negative control for these criteria. Here we computationally and experimentally subject *mass-transfer limited systems*, independent of mechanotransduction, to the purported criteria. The results showed that the negative control (*mass-transfer limited system*) produced the same trends that have been used to identify mechanotransduction-dominant systems. Thus, the widely used viscosity-related shear stress and shear rate criteria are insufficient in determining mechanotransduction-dominant systems. Thus, research should continue to consider the importance of mass transfer in triggering signaling cascades.

## Introduction

Understanding the mechanistic behavior of endothelial signaling pathways is crucial to the search for therapeutic drug delivery targeting vascular diseases. Endothelial signaling pathways continuously respond to varying blood flow parameters and govern downstream DNA synthesis, mRNA transcription, and protein translation. Often generally called, mechanotransduction, these fluid mechanical forces trigger cytoskeletal focal adhesions and subsequent signaling molecules such as Shc-Grb-SOS-Rho-RAS [1]–[3]. However, fluid mechanical forces actually offer two significantly different, but coupled, mechanisms that may impact signaling. These include mechanotransduction and mass transfer. In this more formal description, mechanotransduction consists of the direct interaction of mechanical forces on cellular transducers. Mass transfer, on the other hand, is the result of fluid mechanical forces modifying concentration gradients of signaling chemicals that also impact the subsequent signaling cascade. Both mechanotransduction and mass transfer are significant throughout cellular systems [4]–[7]. However, the underlying factors that stimulate changes in endothelial signaling processes due to fluid mechanical forces remain unclear and have been debated for nearly half-a-century. Schwartz et al. (1995–2011) demonstrated that shear stress is a determinant of endothelial signaling (activated by triggering of focal adhesions), but while assuming that the mechanisms of mechanotransduction is significant, they concede that identifying the mechanism for this process remains unresolved [8]–[10]. Thus a fundamental interest in the mechanism of signaling processes in the endothelium due to fluid mechanics, whether mechanotransduction, mass transfer or both remains an important topic of interest [1]–[10]. Therefore, it remains plausible that fluid-dependent mass transfer may also trigger endothelial signaling cascades.

In order to develop an inferential method for determining the importance of mechanotransduction and mass transfer in endothelial signaling processes, Ando et al. (1988) used viscosity-related tangential flow studies to evaluate signaling results as compared to fluid shear rate and shear stress. The authors used these results to establish criteria for discerning whether mechanotransduction or mass-transfer was dominant in endothelial signaling processes [11]. Their criteria was, if the signaling process was *viscosity-dependent* when plotted against shear rate, but was *viscosity-independent* when plotted against shear stress, then the process was mechanotransduction. While the authors performed both mathematical order-of-magnitude analysis and experiments to arrive at this conclusion, they did not discuss negative controls (i.e., the possibility of not having mechanotransducers or mass transfer). These criteria have been used to conclude the mechanism of fluid mechanical forces on a number of signaling processes [11]–[21].

We evaluate a negative control for the above criteria (a system without mechanotransduction) through both computational modeling and *in-vitro* experiments and evaluated these results to the purported criteria above. We revisit the experiments previously performed by Ando et al. (1988–2009) that have provided the criteria that separate the significance of mechanical transducers and mass transfer on signaling at the endothelium [11]–[21]. We use a negative control where the system is *mass-transfer limited* and is independent of any possible mechanotransducers. We assume that chemical signaling may be proportional to mass transfer of the triggered species, and we directly consider the viscosity dependency for the *mass-transfer limited* systems with respect to shear rate and shear stress in both experimental and computational conditions. To define a negative control consisting of no mechanotransduction, we mathematically simulate a parallel flow chamber experiment to study the mass transfer of triggered species for varying flow parameters. We additionally perform experiments in a non-cellular environment. This eliminates any possible role of biomolecular complexes that could be identified as mechanotransducers. We compare the experimental and simulated *mass-transfer limited* results relative to viscosity dependence with previously published results by Ando et al. (1988–2009) that were used to establish mechanotransduction dependency for endothelial flow-dependent signaling based on the viscosity-dependence criteria [11]–[21].

### Initial Consideration of Mass Transfer and Mechanotransduction in Endothelial Signaling

Fry et al. (1968) showed the effect of velocity gradients on the morphology of endothelial cells by demonstrating the enhanced uptake of Evans blue dye as a result of elevated wall shear rate [22]. Their findings supported the advanced theories that account for shear rate-dependent mass transport by Caro et al. [23]–[24] Caro and Nerem attempted to quantify the transport of ^14^C-4-cholestrol between blood serum and arterial wall in the perfused canine carotid artery [25]. Their results suggested that mass flux might be a possible means of transport of biomolecules between the blood fluid phase and the arterial walls. The authors recommended the necessity for better experimental techniques to ascertain the role of wall shear rate as a plausible mechanism to explain endothelial functions.

In the following years, studies on the endothelial cell function and morphology were performed by a number of researchers [26]–[53]. These authors independently demonstrated that shear stress and blood flow parameters were coupled to the endothelial signaling process.

In 1995, Davies wrote an extensive review where he discussed the mechanotransduction mechanisms that might lead to biochemical, biophysical, and gene regulatory effects of endothelial cells as a direct response to shear stress [54]. The review also suggested the necessity in solving the confounding, yet difficult, problem of decoupling endothelial chemical mass transport from mechanotransduction.

### Establishment of Viscosity-Dependent Criteria for Mechanotransduction Processes

At about the same time, Ando et al. (1988) designed experimental work to differentiate the effects of wall shear stress (τ) from shear rate (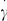) by exploiting their relationship 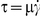
__ [11]. The authors proposed that by altering the viscosity, they could separate the mechanisms of 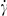
_ _from τ respectively. Endothelial cells were perfused with growth media having high and low viscosities. Ultimately, they established criteria that the significance of mass transport would be exhibited by shear rate viscosity-independency, whereas mechanotransduction would be observed by shear stress viscosity-independency. The authors observed that the increase in intracellular Ca^2+^ was viscosity dependent with varying shear rates and viscosity independent with varying shear stresses. Hence, they concluded that mass transfer of molecules (previously postulated by Fry et al. and Caro et al. [22]–[23]) might not be dominant at the endothelium. Interpreting their results, they concluded that mechanotransduction was the most significant mode of endothelial signaling.

**Figure 1:**
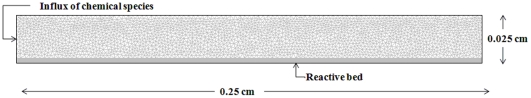
Representation of the quadrilateral boundary layer mesh used for computational modeling of the parallel flow chamber near the region representing the fluid/solid interface. In this example 6776 mesh elements are used with 2500 in the boundary layer. This mesh application improves computational representations of momentum and mass transfer gradients in the boundary layers.

### Consequence of the Viscosity-Dependent Criteria

The work of Ando et al. (1988–2009) led to the search of mechanical transducers or/and mechanical sensors at the endothelium. Since then, several pivotal publications have addressed the significance of mechanotransduction in their in-vitro shear stress experiments and determined the impact of mechanotransducers on mRNA expression, Ca^2+^ influx, lymphocyte adhesion, and cell differentiation [11]–[21]. Although seminal work emerged, the decoupling of mass transfer and mechanotransduction in endothelial signaling pathways was not further addressed [1]–[6], [55]–[63]. However in 1999, Ross again attributed atherosclerosis as a result of both mass transfer and mechanical transduction [64]. Later Ethier suggested the possible role of mass transport in vascular pathologies which was earlier shown not to play an important role through the experiments of Ando et al. (1988) [65], [11].

**Figure 2:**
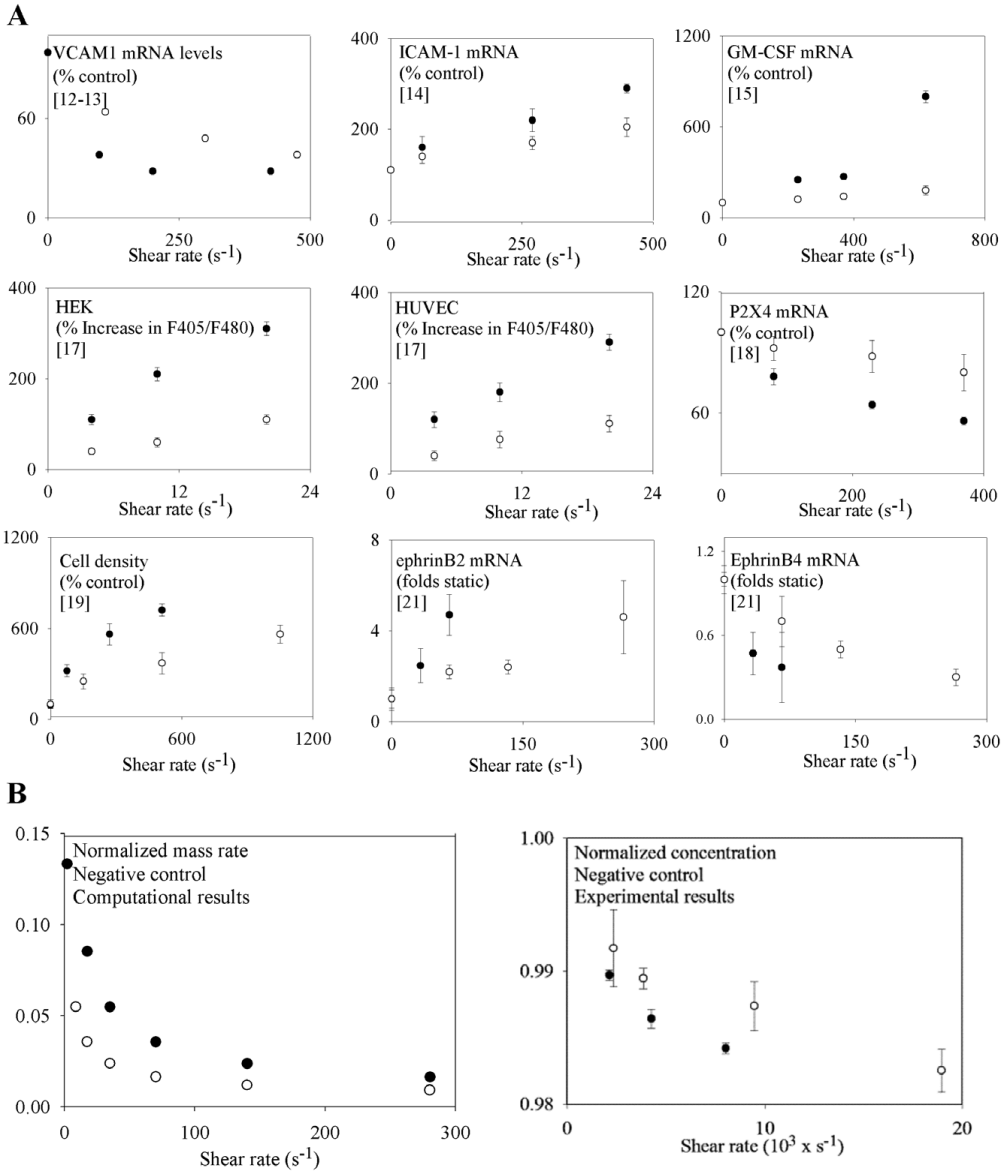
Comparison of viscosity-dependent expression with respect to shear stress from previous in-vitro experimental results and viscosity-dependent mass transfer with respect to shear stress simulation and in-vitro experimental results. The expression of various molecules, cell density, and mRNA levels are shown (A). Note that all the graphs are plotted against shear stress for high viscosity (•) and low viscosity (○) media and demonstrate a similar trend - viscosity independence. This observed viscosity-independent behavior has been previously correlated to mechanotransduction [11]–[21]. However, the results obtained from our computational simulations (normalized average mass rate) and in-vitro flow experiments (normalized average concentration) for mass-transfer limited studies demonstrate similar trends (B).

In recent years, a number of experimental and numerical studies have been carried out to analyze the arterial flow field, flow parameters, and biomolecules that contribute to vascular diseases such as atherosclerosis [66]–[77]. Numerical and mathematical models have been developed for 2D parallel plate flow chambers for steady as well as disturbed flow to study ATP and ADP concentrations [66]–[67]. Further hemodynamic flow has been correlated to endothelial vasoactive agents [68]–[76].

## Materials and Methods

### Ethics Statement

No animals, tissue or cells were used in this study.

### Computer Simulations

#### Problem definition

Simulations are performed using a 2D rectangular flow chamber of dimensions 0.0254 cm × 0.25 cm. Critical dimensions, fluid density, viscosities and maximum velocity are consistent with previously reported work [11]. The length is selected to minimize computational time with fully-developed laminar flow (entry length is 0.027 cm). Wall effects are assumed to be negligible. The apical side of the endothelial cell layer was approximated as a continuous, uniform surface. The mass flux averaged is across the length of the hypothetical endothelial cell layer.

**Figure 3:**
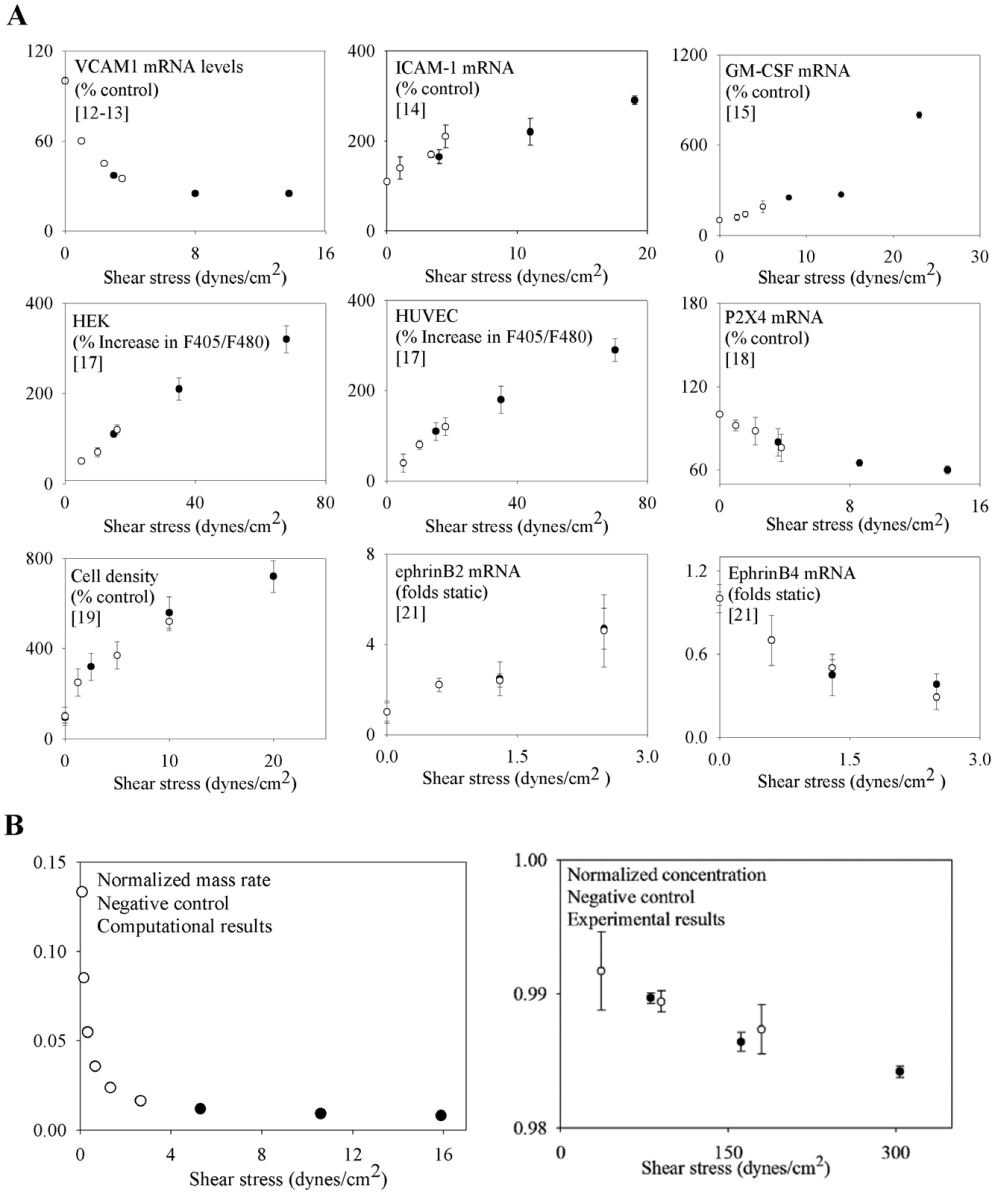
Comparison of viscosity-dependent expression with respect to shear rate from previous in-vitro experimental results and viscosity-dependent mass transfer with respect to shear rate simulation and in-vitro experimental results. The expression of various molecules, cell density, and mRNA levels are shown (A). Note that all the graphs are plotted against shear rate for high viscosity (•) and low viscosity (○) media and demonstrate a similar trend - viscosity dependence. This observed viscosity-dependent behavior has been correlated to mechanotransduction [11]–[21]. However, the results obtained from our computational simulations (normalized average mass rate) and in-vitro flow experiments (normalized average concentration) for mass-transfer limited studies demonstrate similar trends (B).

#### Governing equations

The fluid phase is governed by the continuity equation for incompressible fluid (1) and the Navier-Stokes equation (2). The chemical species flowing through the system follows the equation of conservation of mass and Fick’s Law of Diffusion (3),
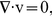
(1)

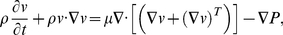
(2)

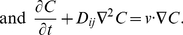
(3)


#### Boundary equations

The transport of biologically active species such as ATP, O_2,_ Ca^2+^, and NO in arteries are considered in this analysis. Similarly, Tarbell et al. (2003) postulated the importance of mass transport for these species in the arteries [71]. In this paper, we analyze the effect of viscosity dependence on transport for species with properties relevant to these biologically active molecules.

The entrance flow is modeled as parabolic and laminar with a species concentration of 1 µM [78]. The diffusivities of the species relative to viscosities are calculated using Stokes-Einstein’s equation as shown in Table 1. Convection and diffusion are selected as the transport mechanism for the species between the fluid and the simulated cell-seeded surface. For ATP, a surface reaction rate constant, *k*, of 1.47×10^−3^ cm·s^−1^ and a diffusion coefficient, *D_B,ATP_*, of 5×10^−6^ cm^2^·s^−1^ are used [66], [72]. The *Da* is calculated as the ratio of the reaction rate to the mass transfer rate,
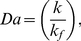
(4)where *k_f_* is the mass transfer coefficient. The estimated Damköhler number of ATP, O_2_ and NO are 17.7, 49 and 173, respectively (Table 1). In addition, the Damköhler number of Ca^2+^ is expected to be directly related to its dynamic relationship with the ADP/ATP concentration at the endothelium [79]–[81]. Recognizing that the apparent surface reaction rate in terms of the bulk concentration is reduced by *1/(1+Da)*, the transport of these small molecular-weight species under these conditions is *mass-transfer limited* as *Da>>1* [39], [82]. Hence a mass-transfer limited boundary condition at the endothelial cell surface is approximated as (5),
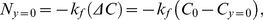
(5)where Co and Cy = 0 are the concentration of the species in the bulk and at the endothelial cell surface, respectively. The scaling parameters for this 2D model are the Reynolds number, Re, and the Da.

**Table 1:** Transport characteristics of O_2_, ATP, and NO in the simulated finite element model.

Parameters	Units	Symbol	Medium 1	Medium 2	Reference
**Density**	g·cm^−1^·s^−1^	ρ	1.024	1.024	[11]
**Kinematic viscosity**	cm^2^·s^−1^	υ	0.0096	0.037	[11]
**ATP diffusivity**	cm^2^·s^−1^	D_ATP_	5.0×10^−6^	1.35×10^−6^	[72] calculated^*^
**ATP mass transfer coefficient**	cm·s^−1^	k_fATP_	1.47×10^−3^	6.02×10^−4^	[72] calculated^**^
**Damkhöler number**		Da_O2_	49	>49	[71]
		Da_ATP_	17.7	>17.7	[71]
		Da_NO_	173	>173	[81]

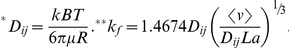

Density, diffusivity, viscosity, mass transfer coefficient, and Damko?ler number for the growth media with the two different viscosities are listed.

Comsol Multiphysics® (Version 3.5, Burlington, MA, USA) is used to numerically simulate the parallel flow chamber. Sparse object oriented linear equations solver (SPOOLES), a library for solving sparse real and complex linear systems of equations, provided by COMSOL Multiphysics® is used to simulate the steady flow model of the parallel plate chamber.

#### Mesh analysis

A mesh independent model shown in Figure 1 is simulated and selected by increasing mesh elements until wall shear stress have a relative error of 0.002%. In order to test the accuracy of the grid size, studies are performed with models having mesh elements of 5,876 and 12,776. A parabolic inlet velocity with *Re = 91* is used for comparing the convergence tolerance. The results imply that the grid size of 6,776 elements is appropriate for this study. Better resolution of the large velocity and concentration gradients at the boundary layers is obtained by implementing a quadrilateral boundary layer mesh that provides a dense element distribution in the normal direction along the boundary where endothelial cells are simulated.

#### Post analysis

The concentration obtained for each mesh element at the inlet, outlet, and the endothelial cell surface is integrated and averaged. The normalized consumed mass rate of species *i* at the endothelial cell surface is calculated using.
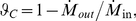
(6)where 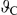, 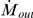, and 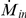 is the normalized mass rate for the consumed species *i*, the mass rate of species *i* flowing out of the chamber, and the mass rate of species *i* flowing into the chamber, respectively. The difference in concentrations across the hypothetical endothelial cell surface was determined to be negligible for any specific case.

### Experimental Method

We experimentally study the effects of flow parameters and fluid properties on an engineered mass transfer system. Mass transfer experiments are performed using an aqueous mixture containing measured quantities of Yellow #5 and #6 dyes, delivered through a rectangular tangential-flow diafiltration module. Water and dye solutions are introduced into the module by two inlets and co-currently delivered on the other side of the module while invoking zero transmembrane pressure. The diafiltration module consists of an upper and lower chamber and is divided by a hydrophilic membrane (Durapore 125 µm, Millipore Inc.).

A separate set of mass transfer experiments introduce an aqueous solution of 40% glycerol and 40% glycerol-dye solution into the engineered mass transfer system. The experimental protocol for glycerol experiments are the same as that of water and dye experiments. The membrane is pre-soaked for ten minutes and the system is primed and pressurized by the corresponding non-dye solution before and after each trial. The dye concentration of the inlet streams for the experiments is 0.25 g/L. Special care is taken while handling the glycerol samples by gently stirring them to obtain spatially uniform concentrations of dye.

Samples are taken at different intervals for a time period of 10 minutes. The concentration of the dye is quantified using a VIS (500 nm) spectrophotometer. SigmaPlot® (Version 10.0.1.25) is used to analyze the data from the spectrophotometer. The resultant mass transport of dye is correlated to varied shear stresses and shear rates. Experiments are performed in triplicate.

## Results and Discussion

### Viscosity-Independent Shear Stress Results

Using a computational fluid dynamics (CFD) model and species mass balance as described in Materials and Methods, we show that a mass-transfer limited model follows the same trends as observed by others who deduced that similar trends indicated mechanotransduction [11]–[21]. The viscosity-independent trends observed in Figure 2: Panel A were previously interpreted to demonstrate the dominance of mechanotransduction at the vascular endothelium. The negative or positive slope is related to the consumption or production of the measured species at the endothelium. As seen in Figure 2: Panel B, the CFD simulation and the *in-vitro* experimental setup using a mass-transfer limited system demonstrate similar viscosity-independent trends.

### Viscosity-Dependent Shear Rate Results

Figure 3 shows the shear rate versus normalized mass flux for two viscosities. Figure 3: Panel A shows the results obtained previously by others through their *in-vitro* experiments [11]–[21]. Again, the negative or positive slope of the measured species is related to its consumption or production at the endothelium. The greater/lower y-axis for higher/lower viscosity media is dependent on whether the reactants or products are analyzed in their respective signaling cascade. Figure 3: Panel B shows the results of the CFD simulations and the *in-vitro* experiments, demonstrating similar viscosity-dependent relationships.

### Differences in Computational and Experimental Model

While comparing and analyzing the results, it is important to consider the differences in the dimensions of the typical parallel plate flow chamber used in our computer simulations (based on Ando et al. (1988)) and the dimensions of the in-house parallel flow chamber used for the experimental results [11]. Despite these differences in dimensions, we observe that the trends observed in both the graphs are similar when the system is mass-transfer limited. More importantly, irrespective of the range of shear rate or shear stress examined, the relevant relationship between viscosity and transport is prevalent across the investigated range for all mass-transfer limited studies and are the same relationships originally used as the criteria for determining whether the transport system was mechanotransduction-dependent. Thus, it appears that the observed trends also scale across dimensions for these flow systems.

### Conclusion

As evident from our results, the criteria of viscosity variation relative to shear rate and shear stress by Ando et al. (1988–2009) are not enough to eliminate mass transfer in determining the signaling mechanism in endothelium processes [11]–[21]. Thus, it remains plausible that biomolecular mass transfer may also be significant in vascular signaling pathways. This work shows that a more cautious analysis that delineates endothelial mechanotransduction from mass transfer remains warranted and that researchers should return to efforts such as those of Caro that considered both mass transfer and mechanotransduction when investigating the endothelial flow-dependent signaling mechanisms [23]–[25], [83].
